# Inhibition of vascular intimal hyperplasia by the myokine Musclin: the role of NPR3/raptor/mTORC1-mediated glycolysis and phenotypic switching of VSMCs: Musclin in intimal hyperplasia

**DOI:** 10.3724/abbs.2025174

**Published:** 2025-10-10

**Authors:** Shanwu Wei, Mi Xiong, De Li, Xiangxiang Deng, Wei Guo, Xiongshan Sun

**Affiliations:** 1 Department of Anesthesiology the General Hospital of Western Theater Command Chengdu 610083 China; 2 Department of Cardiology the General Hospital of Western Theater Command Chengdu 610083 China; 3 Department of Pharmacy the Affiliated Stomatological Hospital of Chongqing Medical University Chongqing 401147 China

**Keywords:** Musclin, vascular intimal hyperplasia, mTORC1, NPR3, glycolysis

## Abstract

Skeletal muscle-derived Musclin exerts multiple effects on the cardiovascular system. However, the role of Musclin in vascular intimal hyperplasia (IH) remains unclear. This study aims to investigate the role and underlying mechanism of Musclin in IH. We overexpress Musclin in skeletal muscle via adeno-associated virus serotype 6 (AAV6)-mediated gene transfer (AAV- *Musclin*) in an injury-induced mouse vascular IH model. Morphological analyses, including hematoxylin and eosin (H&E) staining and Ki-67 immunohistochemistry, are used to evaluate IH severity. Ki-67 immunofluorescence, transwell assay, wound healing assay, and analysis of vascular smooth muscle cell (VSMC) differentiation markers are conducted to assess VSMC phenotypic switching. The extracellular acidification rate (ECAR) assay is utilized to measure glycolysis in VSMCs. Following AAV- *Musclin* transfection, Musclin levels are increased in both skeletal muscle and peripheral blood. Muscle-specific Musclin overexpression ameliorates injury-induced vascular IH. *In vitro*, Musclin represses glycolysis, proliferation, and migration while increasing VSMC differentiation markers in PDGF-BB-stimulated VSMCs. Mechanistically, Musclin inhibits mammalian target of rapamycin complex 1 (mTORC1) activity and induces NPR3-raptor interaction. Restoring mTORC1 activity abolishes the inhibitory effects of Musclin on PDGF-BB-induced VSMC phenotypic switching and its protective role against injury-induced vascular IH. Additionally, *NPR3* silencing abrogates Musclin-mediated suppression of mTORC1 activity, glycolysis, and phenotypic switching in PDGF-BB-treated VSMCs. Collectively, external Musclin supplementation may represent a promising therapeutic strategy for preventing vascular IH-related pathologies.

## Introduction

Vascular intimal hyperplasia (IH) is a key pathological feature of atherosclerosis, coronary in-stent restenosis, and graft failure after coronary artery bypass grafting [1]. Although drug-eluting stents and antiproliferative agents that target IHs have significantly reduced morbidity, restenosis-related complications remain unresolved [2]. IH results from a maladaptive repair response to vascular injury, primarily characterized by abnormal thickening of the intima, phenotypic switching of vascular smooth muscle cells (VSMCs), extracellular matrix deposition, and neointimal formation [3]. As the major component of the vascular wall, VSMC phenotypic switching plays a critical role in IH. The switching process is characterized by excessive proliferation, migration, and dedifferentiation of VSMCs in response to cytokines such as platelet-derived growth factor (PDGF) [4]. Consequently, elucidating novel mechanisms underlying VSMC phenotypic switching is essential for preventing vascular IH.

Physical exercise significantly reduces the morbidity and mortality of cardiovascular diseases (CVDs), such as coronary heart disease [5]. The beneficial effects of exercise on the cardiovascular system are largely mediated by skeletal muscle-derived myokines [6]. Several myokines are known to regulate VSMC function and vascular diseases. For example, irisin inhibits the pyroptosis and osteoblastic transformation of VSMCs [ 7, 8] . Elevated myostatin suppresses VSMC proliferation and inhibits vascular restenosis [9]. Musclin, an exercise-induced myokine, has been implicated in several CVDs, including hypertension, heart failure, and pulmonary hypertension [ 6, 10, 11] . Additionally, Musclin exerts anti-proliferative effects on cell types such as fibro-adipogenic progenitors [12]. However, the role of Musclin in VSMC phenotypic switching and vascular IH remains largely unknown.

Musclin has been shown to inhibit glucose metabolism in skeletal muscle, adipose tissue, and pulmonary arteries [ 11, 13, 14] . Enhanced glucose metabolism is closely linked to excessive proliferation and migration of VSMCs, as well as vascular IH [15]. Consequently, glycolysis may represent a potential mechanism through which Musclin affects VSMCs. In response to growth factors, energy shifts, and pathological stress, mammalian target of rapamycin (mTOR) functions as a central signal that regulates intracellular metabolism by forming complexes, including mTOR complex 1 (mTORC1) and mTORC2 [16]. mTORC1 promotes glucose uptake and glycolysis, thereby driving VSMC proliferation, migration, and vascular IH [17]. However, whether mTORC1-mediated glucose metabolism acts downstream of Musclin to regulate VSMC phenotypic switching and vascular IH remains unclear.

In the present study, adeno-associated virus serotype 6 (AAV6)-mediated skeletal muscle *Musclin* gene transfer (AAV- *Musclin*) increased Musclin levels in muscle and peripheral blood. We demonstrated that *in vivo* Musclin overexpression alleviates vascular IH. Furthermore, Musclin significantly suppressed PDGF-BB-induced VSMC proliferation, migration, and dedifferentiation. Mechanistically, Musclin exerts its inhibitory effects on VSMCs through reduced glucose metabolism resulting from the suppression of mTORC1 activation. Additionally, the membrane receptor natriuretic peptide receptor 3 (NPR3) was identified as essential for Musclin-induced mTORC1 inactivation in VSMCs. These findings provide crucial insights for the development of therapeutic strategies against vascular IH.

## Materials and Methods

### Animal experiments

All experimental procedures were approved by the Institutional Animal Care and Use Committee and the Ethics Committee of the General Hospital of Western Theater Command. Smooth muscle cell-specific Tuberous sclerosis complex ( *Tsc1*)-knockdown ( *Tsc1* ^KD^) mice on a C57BL/6J genetic background were obtained from Jackson Laboratories (West Grove, USA). Control C57BL/6J mice were purchased from Dashuo Animal Science and Technology (Chengdu, China). All the mice were housed under a 12/12-h day/night cycle at room temperature with free access to water and food. The mouse carotid artery injury model was established as previously described [17]. Briefly, the mice were anaesthetized via an intraperitoneal injection of pentobarbital (40 mg/kg). The left common carotid artery and left internal carotid artery were exposed and temporarily occluded, while the left external carotid artery was permanently ligated. A metal guidewire was inserted into the left common carotid artery and rubbed back and forth to denude the endothelium. Blood flow was then restored to the left common carotid artery and left internal carotid artery. On postoperative day 14, the mice were anaesthetized via decapitation under deep anesthesia (pentobarbital, 100 mg/kg, i.p.). Artery tissues were harvested for subsequent analysis.

### AAV- *Musclin* transfection *in vivo*

The *Musclin* cDNA was tagged with Myc and integrated downstream of the muscle creatine kinase promoter into the pdsMCKE vector. Musclin overexpression *in vivo* was achieved by intramuscular injection of AAV- *Musclin* (5×10 ^11^ pfu/mL; 50 μL) administered immediately post-injury.

### Western blot analysis and co-immunoprecipitation (co-IP) assay

Protein lysates were prepared from quadriceps muscle, arteries, or cultured VSMCs using RIPA buffer (Beyotime, Shanghai, China). Western blot analysis was performed as described in our previous study [17]. In brief, lysates were electrophoresed on 8%–12% sodium dodecyl sulfate-polyacrylamide gels and transferred to 0.45-μm polyvinylidene fluoride membranes (Millipore, Billerica, USA). The membranes were blocked with 5% bovine serum albumin for 1 h, incubated overnight with the corresponding primary antibodies at 4°C, and then incubated with the corresponding secondary antibodies. The primary antibody against Musclin was obtained from BioVendor (Brno, Czech Republic). The primary antibodies against α-SMA and NPR3 were obtained from Abcam (Cambridge, UK). The primary antibodies against calponin 1, phosphor (p)-S6, S6, phosphor (p)-eukaryotic translation initiation factor 4E-binding protein 1 (4EBP1), 4EBP1, TSC1, raptor, and β-actin were purchased from Cell Signaling Technology (CST; Danvers, USA). For the co-IP assay, the immunoprecipitant (IP) and input lysates were analyzed by western blot analysis using anti-NPR3 and anti-raptor antibodies.

### Enzyme-linked immunosorbent assay (ELISA) of Musclin level in peripheral blood

The musclin level in peripheral blood was measured using a mouse Osteocrin ELISA kit (Biomatik, Kitchener, Ontario, Canada) according to the manufacturer’s instructions.

### Immunohistochemical staining

Morphological analysis of carotid arteries was performed via hematoxylin and eosin (H&E) staining and Ki-67 immunohistochemistry as previously described [17]. Briefly, arterial tissues were cut into 4-μm sections. For H&E analysis, the sections were stained with hematoxylin and eosin. For Ki-67 staining, the sections were incubated overnight at 4°C with an anti-Ki-67 primary antibody (1:500; Bioss, Beijing, China), incubated with a corresponding secondary antibody, and finally counterstained with Mayer’s hematoxylin. Images were acquired and analyzed using Image-Pro Plus software (NIH, Bethesda, USA).

### Culture and treatment of VSMCs

Primary VSMCs were isolated from the thoracic aortas of 8–10-week-old C57BL/6J mice as previously described [17]. After the endothelia and adventitia were removed, the arteries were minced and digested with 0.25% trypsin (HyClone, Carlsbad, USA). The VSMCs were cultured in complete medium (HyClone) for further use. Recombined PDGF-BB (30 ng/mL; R&D Systems, Minneapolis, USA) was used to treat VSMCs for 24 h. VSMCs were incubated with Musclin (50 nM; Abcam) for 24 h [ 10, 18] . For siRNA transfection, VSMCs were incubated for 8 h with *Tsc1*i or *Npr3*i and Lipofectamine RNAiMAX Transfection Reagent (Invitrogen, Carlsbad, USA). the sequence of *Tsc1*i is 5′-GCUUUGACUCUCCCUUCUA-3′ [19]; the sequence of *Npr3*i is 5′-GCUCUACAGCGACGACAAA-3′ [10]; and the sequence of NC is 5′-UGGUUUACAUGUCGACUAA-3′.

### Immunofluorescence staining

Immunofluorescence staining for Ki-67 and α-SMA was performed as previously described [17]. VSMCs were fixed, blocked with 5% BSA, and incubated overnight at 4°C in the dark with primary antibodies against Ki-67 (1:1000; CST) and α-SMA (1:2000; CST). After washing, the VSMCs were incubated for 1 h with Alexa Fluor 488-conjugated goat anti-rabbit and Alexa Fluor 594F (ab’)-conjugated goat anti-mouse secondary antibodies (1:2500; Molecular Probes Inc., Eugene, USA). The nuclei were counterstained with DAPI (5 mg/mL; VECTOR Labs, Burlingame, USA) at room temperature for 5 s. Images were acquired via an immunofluorescence microscope (MPS 60; Leica, Wetzlar, Germany).

### Transwell assay

VSMCs were seeded into the upper chamber, which was equipped with an 8-μm pore size insert (Millipore). The lower chamber was filled with medium containing or lacking PDGF-BB (30 ng/mL). After 8 h of incubation, non-migrated VSMCs inside the upper chamber were removed, while migrated VSMCs outside the upper chamber were fixed and stained with 1% crystal violet solution for 20 min. Migrated cells were quantified by counting five random fields per chamber.

### Wound healing assay

VSMCs were seeded into 6-well plates (1 × 10 ^5^ cells/well). After cell attachment, a scratch was created in the VSMC monolayer using a 200-μL sterile pipette. Wound healing rates were quantified through microscopic visualization.

### RNA sequencing data analysis

PDGF-BB-treated VSMCs transfected with Musclin or vehicle were used for RNA sequencing. Differentially expressed genes (DEGs) identified via RNA-sequencing analysis were screened via the R package DESeq2 (v 1.38.3) [20]. *P*  < 0.05 and a fold change ≥ 1.5 were considered the cut-offs for DEGs.

### Functional enrichment analysis

Gene set variation analysis (GSVA) was used to calculate the pathway activity score for each sample on the basis of KEGG pathway gene sets from MSigDB by the R package GSVA [21]. The R package limma was used to screen the differential GSVA scores.

### Extracellular acidification rate (ECAR) analysis

An ECAR assay was performed to assess glycolytic alternations as previously described [17]. Briefly, treated VSMCs were seeded in a Seahorse 96-well plate and sequentially incubated with glucose, oligomycin (oxidative phosphorylation inhibitor), or 2-deoxy-D-glucose (2-DG, glycolytic inhibitor) according to the manufacturer’s instructions (MCE, Shanghai, China). Basal glycolysis was calculated as follows: (the last rate measurement before oligomycin application)-(minimum rate measurement before glucose addition). Glycolytic capacity was defined as (maximal rate measurement after oligomycin application)–(minimum rate measurement after 2-DG addition).

### Statistical analysis

Unpaired Student’s *t* test was used for statistical analysis to compare 2 independent groups. Two-way analysis of variance (ANOVA) was applied for statistical analysis to compare two factors with corresponding *post hoc* tests. Samples following a normal distribution were compared via the LSD test; otherwise, they were analyzed via Dunnett’s T3 test. Data are presented as the mean ± SD. *P < *0.05 was considered statistically significant.

## Results

### Increased expression of Musclin in skeletal muscle attenuates IH in mouse carotid arteries

**Figure 1:**
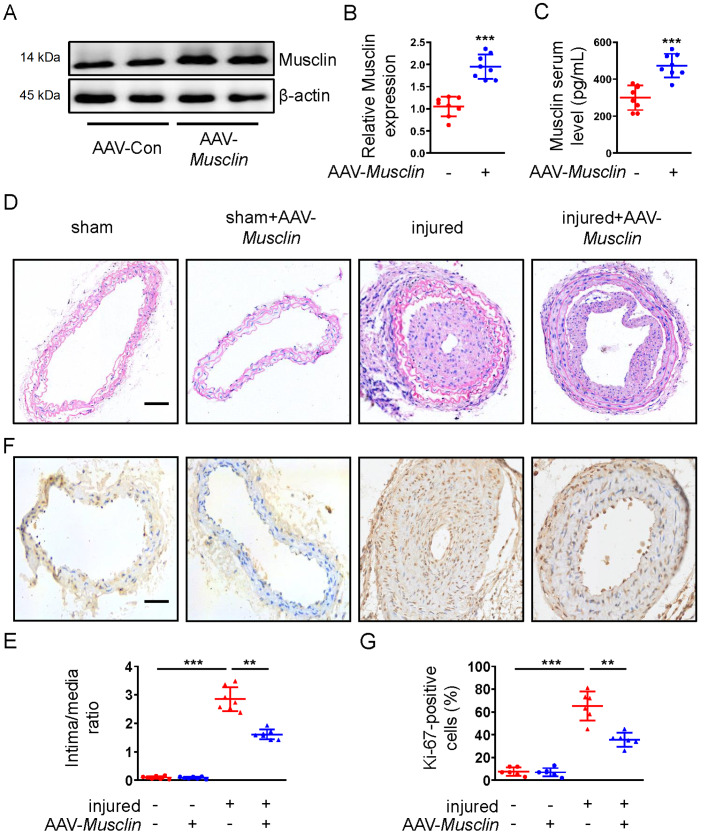
Figure 1 Increased expression of Musclin in skeletal muscle attenuates vascular intimal hyperplasia (IH) in injured mouse arteries (A,B) Representative western blot bands and quantitative analysis of Musclin levels in the right quadricep muscle across experimental groups (n = 8). (C) ELISA quantification of Musclin in peripheral blood from each group (n = 8). (D,E) Representative H&E-stained cross-sections (scale bar = 50 μm) of mouse arteries and the corresponding intima-to-media for each group (n = 7). (F,G) Representative Ki-67 immunohistochemical staining (scale bar= 50 μm) of arteries and quantitative analysis of the percentage of Ki-67-positive cells within the neointima (n = 6). **P < 0.01, ***P < 0.001. Student’s t test for (B,C). Post hoc test for Dunnett’s T3 test for (E) and (G).

A carotid artery injury model was used to investigate the impact of Musclin on vascular IH in adult mice. We employed AAV- *Musclin* carrying the muscle creatine kinase promoter to increase Musclin levels via intramuscular injection immediately post-injury. On day 14, Musclin levels in the quadriceps muscle and peripheral blood were significantly increased ( Figure 1A–C), indicating successful secretion of Musclin into the circulation. Injured carotid arteries exhibited marked increases in the medial area, intimal area, and intima/media ratio ( Figure 1D,E), as well as elevated Ki-67-positive cells within the neointima ( Figure 1F,G), indicating injury-induced VSMC proliferation and IH. Conversely, compared with control mice, mice with exogenous Musclin expression in skeletal muscle presented markedly reduced medial and intimal areas, intima/media ratios, and intimal Ki-67-positive cells ( Figure 1D–G). However, Musclin overexpression had no effect on uninjured carotid arteries.

### Musclin inhibits PDGF-BB-induced phenotypic switching in VSMCs

**Figure 2:**
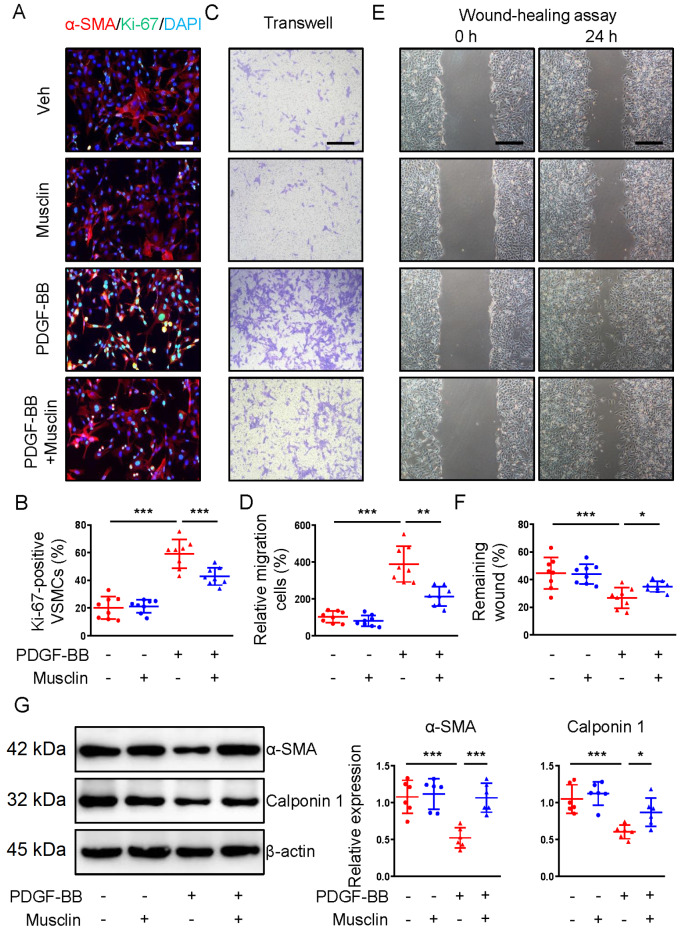
Figure 2 Musclin inhibits PDGF-BB-induced phenotypic switching in VSMCs (A) VSMCs were treated with either vehicle or Musclin (50 nM) in the presence or absence of PDGF-BB (30 ng/mL) for 24 h, followed by immunofluorescence staining for α-SMA (red), Ki-67 (green), and DAPI (blue). Representative images of Ki-67 staining are shown (scale bar = 50 μm). (B) Percentage of Ki-67-positive VSMCs per group (n = 8). (C,E) Migration of VSMCs was assessed using transwell and wound healing assays (scale bar = 200 μm). Representative images are shown. (D) Quantification of migrated VSMCs per group (n = 8). (F) Wound healing rates across groups (n = 8). (G) Representative western blot bands and quantitative analysis of α-SMA and Calponin 1 expression in VSMCs from each group (n = 6). *P < 0.05, **P < 0.01, and ***P < 0.001. Post hoc test for LSD in (B,F,G). Post hoc test for Dunnett’s T3 test in (D).

VSMC phenotypic switching, characterized by aberrant VSMC proliferation, migration, and loss of differentiation markers, is a critical event in vascular IH [1]. To determine whether skeletal muscle-derived Musclin influences this process, we utilized PDGF-BB, a key mediator of VSMC phenotypic switching in vascular IH [4], to assess the effect of Musclin *in vitro*. PDGF-BB significantly increased VSMC proliferation, as evidenced by increased numbers of Ki-67-positive cells. However, this effect was markedly attenuated by Musclin treatment ( Figure 2A,B). Cell migration was assessed via Transwell and wound healing assays. PDGF-BB robustly increased VSMC transmigration in the control group, whereas Musclin treatment significantly blunted this effect ( Figure 2C,D). Concurrently, PDGF-BB accelerated wound closure in control VSMCs, which was also suppressed by Musclin ( Figure 2E,F).

PDGF-BB is known to cause VSMC dedifferentiation, characterized by reduced expression of contractile markers, such as α-SMA and calponin 1 [ 17, 22] . We observed significant suppression of α-SMA and calponin 1 protein expressions in PDGF-BB-treated VSMCs, which was reversed by Musclin treatment ( Figure 2G). Notably, Musclin did not affect the proliferation, migration, or differentiation of PDGF-BB-free VSMCs. Since the level of circulating Musclin *in vivo* is far lower than that *in vitro*, Musclin might be considered a potential pharmacological agent against IH rather than a physiological regulator *in vivo*. Collectively, these results suggest that Musclin specifically suppresses PDGF-BB-induced phenotypic switching without affecting basal VSMC function.

### Musclin inhibits glycolysis and phenotypic switching in PDGF-BB-treated VSMCs by reducing mTORC1 activity

**Figure 3:**
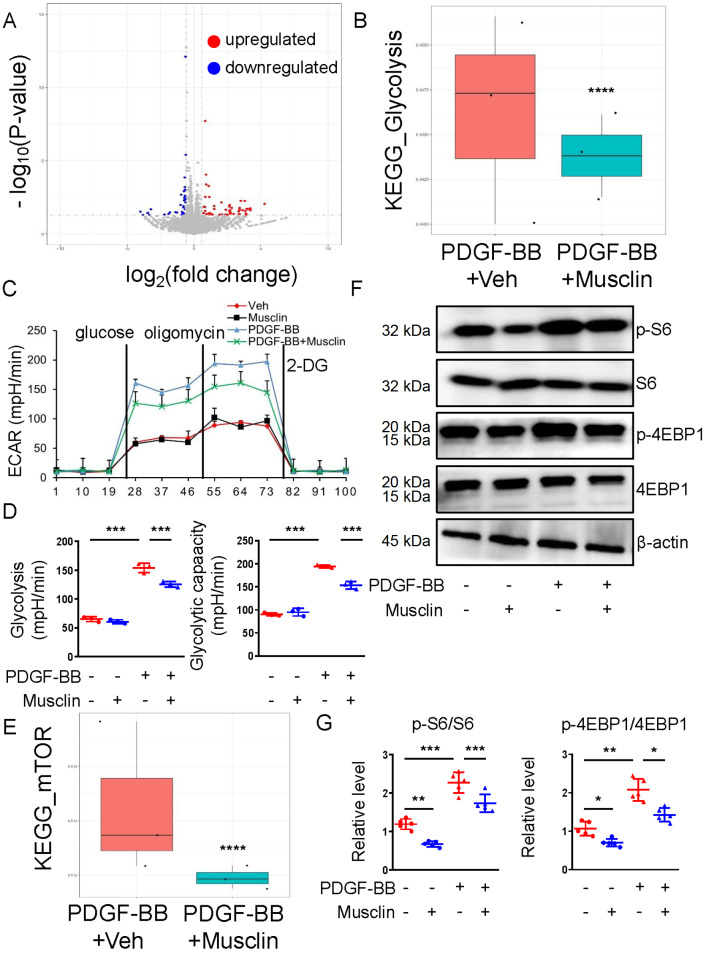
Figure 3 Musclin suppresses glycolysis and mTORC1 activity in PDGF-BB-stimulated VSMCs (A) Volcano plot showing genes that are differentially expressed between the PDGF-BB + vehicle and PDGF-BB + Musclin groups. Red and blue points represent significantly up- and down-regulated genes, respectively; gray points indicate nonsignificant changes. (B) Box plot comparing GSVA enrichment scores for the KEGG glycolysis pathway between groups. (C,D) Extracellular acidification rate (ECAR) measurements showing glycolysis and glycolytic capacity in VSMCs from each group (n = 3). (E) Box plot comparing GSVA enrichment scores for the KEGG mTOR signaling pathway. (F,G) Representative western blot bands and quantitative analysis of p-S6/S6 and p-4EBP1/4EBP1 ratios in VSMCs across groups (n = 5). *P < 0.05, **P < 0.01, ***P < 0.001, and ****P < 0.0001. Post hoc test for LSD in (D,G) (p-S6/S6). Post hoc test for Dunnett’s T3 test in (G) (p-4EBP1/4EBP1).

The mechanisms underlying PDGF-BB-induced VSMC phenotypic switching involve multiple factors. We therefore performed RNA sequencing on PDGF-BB-stimulated VSMCs with or without Musclin treatment. The results revealed alterations in several glucose metabolism-related genes in PDGF-BB-stimulated VSMCs after Musclin treatment ( Figure 3A). Subsequent GSVA revealed that Musclin treatment suppressed glycolytic pathway activity ( Figure 3B). Given that metabolic switching toward glucose metabolism drives VSMC phenotypic transition [23], we assessed glycolytic function via the ECAR. Consistent with the transcriptomic data, PDGF-BB increased the glycolytic capacity of VSMCs, which was attenuated by Musclin ( Figure 3C,D). These findings indicate that Musclin suppresses phenotypic switching through glycolysis inhibition.

GSVA revealed a negative correlation between Musclin treatment and mTOR pathway activity in VSMCs ( Figure 3E). As a central signaling hub responsive to various stimuli, including cytokines and growth factors, mTORC1 critically regulates glycolysis and phenotypic switching in VSMCs [17]. To investigate whether mTORC1 mediates Musclin-mediated VSMC regulation, we assessed mTORC1 activity in PDGF-BB-stimulated VSMCs following Musclin treatment. Musclin significantly attenuated PDGF-BB-induced mTORC1 activation, as evidenced by reduced phosphorylation of its key effectors S6 and 4EBP1 ( Figure 3F,G).

**Figure 4:**
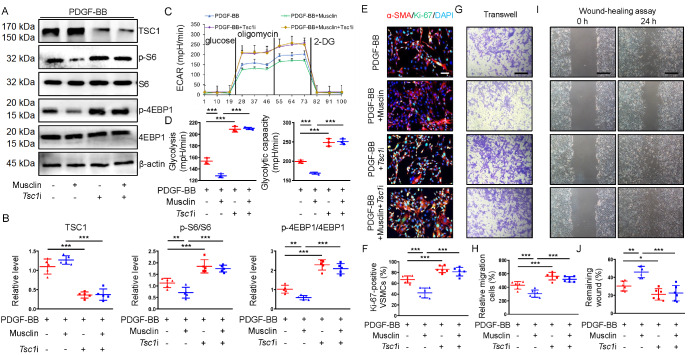
Figure 4 Musclin suppresses glycolysis, proliferation, and migration in PDGF-BB-challenged VSMCs by reducing mTORC1 activity (A,B) Representative western blot bands and quantitative analysis of TSC1, p-S6/S6 and p-4EBP1/4EBP1 in VSMCs from each group (n = 5). (C,D) Glycolysis and glycolytic capacity measured by the ECAR in VSMCs across groups (n = 3). (E,F) Immunofluorescence staining of VSMCs for α-SMA (red), Ki-67 (green), and DAPI (blue). Representative images of Ki-67 staining (scale bar = 50 μm) and the percentage of Ki-67-positive VSMCs are shown (n = 6). (G,H) Migration of VSMCs was assessed via a transwell assay (scale bar = 200 μm), and the results were quantified (n = 6). (I,J) Wound healing rates of VSMCs from each group (scale bar = 200 μm; n = 6). *P < 0.05, **P < 0.01, and ***P < 0.001. Post hoc test for LSD in (B,D,F,H,J).

Tuberous sclerosis 1 (TSC1) is a canonical upstream inhibitor of mTORC1 [24]. We therefore transfected VSMCs with *Tsc1* siRNA ( *Tsc1*i) to reactivate mTORC1. In this study, silencing of *TSC1* restored mTORC1 activity in PDGF-BB-challenged VSMCs ( Figure 4A,B). We next examined whether mTORC1 reactivation abrogates Musclin-mediated suppression of glycolysis and phenotypic transition. ECAR analysis revealed that *Tsc1*i enhanced the glycolytic capacity of PDGF-BB-challenged VSMCs, which was not affected by Musclin ( Figure 4C,D). Furthermore, *TSC1* silencing abolished Musclin-mediated inhibition of proliferation and migration in PDGF-BB-treated VSMCs ( Figure 4E–J), indicating that mTORC1 inhibition is essential for musclin-mediated regulation of glycolysis and phenotypic switching.

### Skeletal muscle-derived Musclin inactivates mTORC1 to attenuate glucose utilization and vascular IH

**Figure 5:**
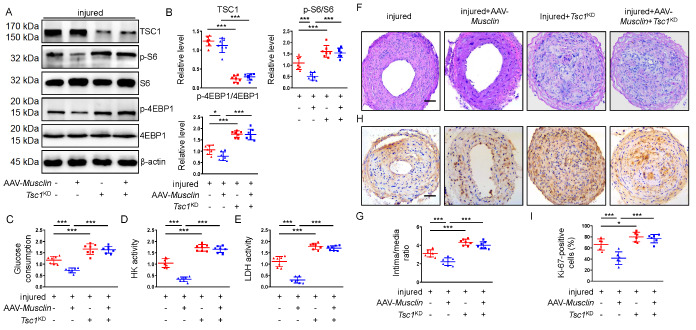
Figure 5 Skeletal muscle-derived Musclin alleviates vascular IH by reducing mTORC1 activity (A,B) Representative western blot bands and quantitative analysis of TSC1, p-S6/S6, and p-4EBP1/4EBP1 in arteries from each group (n = 7). (C–E) Glucose consumption (C), HK activity (D), and LDH activity (E) in arterial tissue across groups (n = 7). (F,G) Representative H&E-stained arterial sections (scale bar = 50 μm) and corresponding intima-media ratios (n = 7). (H,I) Representative Ki-67 staining of arteries (scale bar = 50 μm) and quantitative analysis of Ki-67-positive cells within the neointima (n = 6). *P < 0.05, ***P < 0.001. Post hoc test for LSD in (B–D,G,I). Post hoc test for Dunnett’s T3 test in (E).

Aberrant vascular mTORC1 activation drives pathological vascular remodeling [24]. We therefore investigated whether mTORC1 inhibition is essential for Musclin-mediated vascular protection against IH. Protein analysis revealed reduced phosphorylation of S6 and 4EBP1 in injured arteries following exogenous Musclin expression ( Figure 5A,B), indicating that Musclin suppresses vascular mTORC1 activity post-injury. Using smooth muscle-specific *Tsc1-*knockdown ( *Tsc1* ^KD^) mice, we observed markedly enhanced mTORC1 activity in injured arteries ( Figure 5A,B). Importantly, Musclin did not attenuate mTORC1 activity in the injured arteries of *Tsc1* ^KD^ mice.

Hexokinase (HK) and lactate dehydrogenase (LDH), the rate-limiting enzymes for glycolysis and lactate dehydrogenation, respectively, are key indicators of glycolytic activity [25]. In injured arteries, Musclin overexpression significantly reduced glucose consumption and HK/LDH activity. However, these inhibitory effects of Musclin on the glycolytic metric were abolished in *Tsc1* ^KD^ mice ( Figure 5C–E). Histological analysis revealed that *TSC1* knockdown exacerbated injury-induced vascular IH. Notably, Musclin overexpression failed to inhibit this mTORC1 reactivation-driven increase in IH ( Figure 5 F–G). Ki-67 staining revealed a significant increase in the number of proliferating cells upon mTORC1 hyperactivation, which remained unaffected by Musclin treatment ( Figure 5H,I). These results demonstrate that Musclin protects against vascular IH and suppresses glucose metabolism *in vivo* through mTORC1-dependent mechanisms.

### Musclin inactivates mTORC1 via NPR3-raptor interaction and raptor recruitment in PDGF-BB-stimulated VSMCs

**Figure 6:**
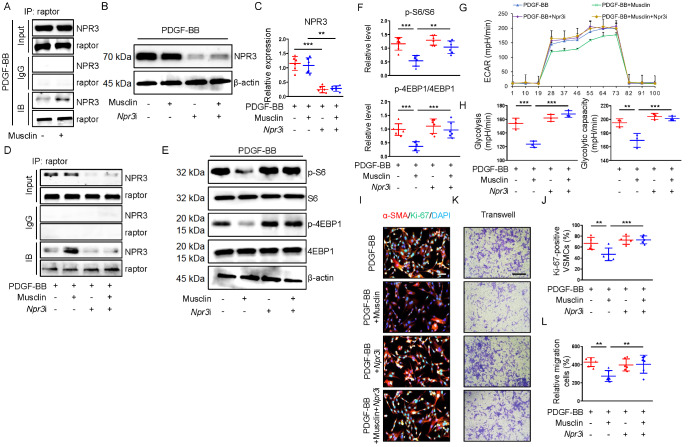
Figure 6 Musclin inhibits mTORC1 signaling in PDGF-BB-stimulated VSMCs by promoting NPR3-raptor interaction (A) Coimmunoprecipitation (co-IP) assay of VSMCs treated with or without Musclin (50 nM, 24 h). (B,C) Representative western blot bands and quantitative analysis of NPR3 expression in VSMCs across groups (n = 6). (D) Co-IP analysis of VSMCs from each group. (E,F) Western blot bands and quantification of p-S6/S6 and p-4EBP1/4EBP1 in VSMCs from each group (n = 6). (G,H) Glycolysis and glycolytic capacity measured by the ECAR in VSMCs (n = 3). (I,J) Immunofluorescence staining for α-SMA (red), Ki-67 (green), and DAPI (blue). Representative images of Ki-67 staining (scale bar = 50 μm) and the percentage of Ki-67-positive VSMCs are shown (n = 5). (K,L) Migration of VSMCs was assessed by a transwell assay (scale bar = 200 μm), and the results were quantified (n = 6). **P < 0.01, and ***P < 0.001. Post hoc test for LSD in (F,H,J,L). Post hoc test for Dunnett’s T3 test in (C).

As a paracrine myokine, Musclin may regulate mTORC1 activity through receptor binding. NPR3 is a well-known membrane receptor for Musclin [10], but its relationship with mTORC1 remains unclear. We first determined whether NPR3 directly interacts with mTORC1. Co-IP revealed that Musclin promotes the recruitment of raptor, the core component of mTORC1, to NPR3 in PDGF-BB-stimulated VSMCs without altering raptor expression ( Figure 6A). These findings suggest that Musclin modulates mTORC1 activity via NPR3 binding and subsequent NPR3-raptor interaction. Using *Npr3*i to silence *NPR3* in PDGF-BB-treated VSMCs ( Figure 6B,C), we found that Musclin did not alter NPR3 expression but required NPR3 for its effects: *NPR3* knockdown abolished the Musclin-induced NPR3-raptor interaction and mTORC1 dephosphorylation ( Figure 6D–F).

We next validated the role of NPR3 in the Musclin-mediated effects on PDGF-BB-stimulated VSMCs. A previous study demonstrated that *NPR3* knockout enhances insulin-stimulated glucose uptake in adipocytes [26]. Similarly, *NPR3* silencing reversed Musclin-mediated inhibition of glycolysis in PDGF-BB-stimulated VSMCs, as shown by ECAR analysis ( Figure 6G,H). Although reduced NPR3 expression is correlated with increased human VSMC proliferation [27], its precise role remains unknown. Notably, *NPR3* knockdown alone did not affect PDGF-BB-induced proliferation or migration without Musclin treatment. However, it completely abolished Musclin-mediated suppression of these processes ( Figure 6I–L). Thus, Musclin represses mTORC1 activity in PDGF-BB-stimulated VSMCs by binding to NPR3 and subsequently promoting recruitment.

## Discussion

**Figure 7:**
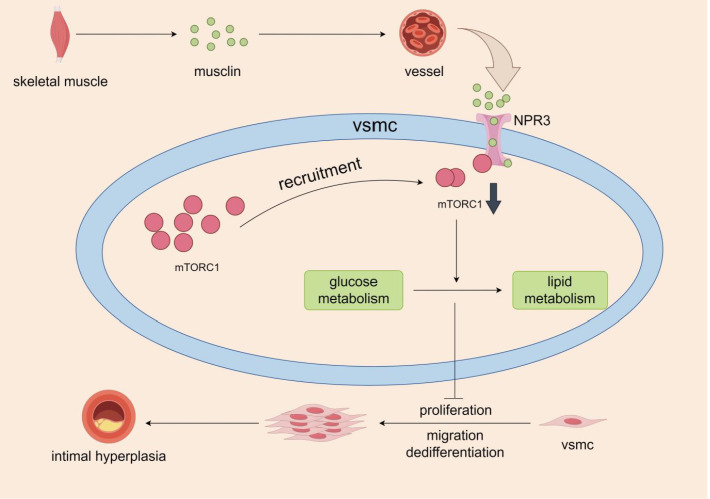
Figure 7 Skeletal muscle-derived Musclin attenuates vascular IH by inhibiting VSMC glycolysis and phenotypic switching via an NPR3/raptor/mTORC1-dependent mechanism Overexpression of Musclin in skeletal muscle leads to its secretion into the circulation. Circulating Musclin binds to NPR3 on VSMCs, inducing an interaction between NPR3 and raptor, which inactivates mTORC1 signaling. Consequently, mTORC1 inhibition suppresses glycolysis and phenotypic switching in VSMCs, thereby attenuating vascular IH. This figure is created by Figdraw.

In this study, we identified Musclin as an exercise-responsive myokine that protects against vascular IH by suppressing mTORC1 activity, glycolysis, and phenotypic switching in VSMCs. Mechanistically, Musclin binds to its receptor NPR3, induces NPR3-raptor interaction, and thereby inactivates mTORC1 in PDGF-BB-stimulated VSMCs. Critically, reactivating mTORC1 rescued Musclin-mediated suppression of vascular IH and VSMC phenotypic switching. These findings establish the critical role of Musclin in regulating the NPR3/raptor/mTORC1 signaling axis to mitigate pathological vascular remodeling ( Figure 7).

Exercise plays a critical role in maintaining cardiovascular health, including attenuating VSMC phenotypic transition [ 28, 29] . Myokines are key mediators through which exercise confers cardiovascular protection. As an exercise-responsive myokine, Musclin is recognized primarily as a regulator of bone development and formation [30]. Emerging evidence indicates that Musclin also critically influences vascular pathophysiology: decreased circulating Musclin correlates with poor prognosis in hypertensive patients and those undergoing transcatheter aortic valve implantation [ 6, 31] . Furthermore, Musclin alleviates vascular inflammation by inhibiting endothelial-monocyte adhesion and inflammatory cytokine expression [18]. Consistent with these reports, our study revealed that Musclin protects against vascular IH. Specifically, skeletal muscle-derived Musclin significantly reduced neointimal formation and decreased the number of proliferating cells within the neointima.

We next explored the mechanism by which Musclin prevents injury-induced vascular IH. Phenotypic switching of VSMCs, characterized by increased VSMC proliferation/migration and decreased VSMC differentiation, plays a central role in the development of vascular IH [4]. *In vitro*, Musclin inhibited PDGF-BB-induced VSMC proliferation, aligning with its known anti-proliferative effects on fibro-adipogenic progenitors [12]. Migration assays confirmed that Musclin attenuated PDGF-BB-stimulated VSMC migration. Furthermore, Musclin reversed PDGF-BB-induced dedifferentiation, as evidenced by the restored expression of VSMC contractile markers. Mechanistically, Musclin downregulates glucose metabolism-related genes. Given that a metabolic shift toward glycolysis drives VSMC phenotypic switching [ 15, 19] , we found that Musclin suppressed PDGF-BB-induced glycolysis *in vitro*. Consistently, *in vivo* Musclin overexpression inhibited vascular glucose utilization during IH development. These data demonstrate that Musclin confers vascular protection through glycolysis inhibition and phenotypic switching suppression in VSMCs.

Next, we investigated the mechanism of Musclin in VSMCs. GSVA revealed significant modulation of the mTOR pathway by Musclin in PDGF-BB-stimulated VSMCs. mTORC1, a major complex of mTOR, orchestrates cellular processes, including proliferation, migration, mitochondrial function, and autophagy, through its downstream effectors S6 and 4EBP1 [32]. Crucially, aberrant mTORC1 activation drives glycolysis, VSMC phenotypic switching, and vascular IH [ 17, 19] . To validate the role of mTORC1 in Musclin-mediated regulation, we silenced *TSC1* to reactivate mTORC1. This intervention abolished the inhibitory effects of Musclin on glycolysis, proliferation, and migration in PDGF-BB-treated VSMCs. Consistent with these findings *in vivo*, Musclin overexpression failed to reduce vascular glucose utilization or attenuate injury-induced vascular remodeling in *Tsc1* ^KD^ mice. Our study thus establishes mTORC1 inhibition as the core mechanism underlying Musclin-mediated vascular protection.

As a muscle-synthesized secretory protein, Musclin functions by binding to its specific receptors. NPR3 is a canonical membrane receptor for Musclin [27]. To explore whether NPR3 directly mediates Musclin’s regulation of mTORC1, we used a co-IP assay to assess the interaction between NPR3 and raptor, a core component of mTORC1. The results showed that Musclin significantly enhanced the NPR3-raptor interaction in PDGF-BB-treated VSMCs, suggesting that Musclin-induced direct binding of mTORC1 to NPR3 may inactivate mTORC1. Consequently, we silenced *NPR3* and observed that both the NPR3-raptor interaction and Musclin-induced mTORC1 inactivation were abolished. Previous studies revealed that NPR3 negatively regulates VSMC proliferation and elevates blood pressure [27]. However, whether the NPR3/mTORC1 axis contributes to Musclin-mediated effects on VSMCs remains unclear. In this study, NPR3 blockade abolished the suppressive effects of Musclin on glycolysis, proliferation, and migration in PDGF-BB-stimulated VSMCs. Notably, *NPR3* silencing alone had little effect on PDGF-BB-induced VSMC glycolysis, proliferation, and migration without Musclin treatment. This result seems slightly inconsistent with previous literature [27]. This may be because PDGF-BB treatment of VSMCs results in high mTORC1 activity, and in the absence of Musclin, NPR3-raptor binding is relatively weak. Under these conditions, *NPR3* knockdown may not be sufficient to further enhance PDGF-BB-induced VSMC proliferation and migration by affecting mTORC1 activity. This observation does not contradict the finding that *NPR3* knockdown affects the proliferation and migration of PDGF-BB-free VSMCs, as described in a previous study [27]. However, after treatment with Musclin, the ability of NPR3 to bind to and sequester raptor was significantly enhanced, thereby suppressing mTORC1 activity in PDGF-BB-stimulated VSMCs. In this context, *NPR3* knockdown markedly antagonized the inhibitory effects of Musclin on PDGF-BB-induced VSMC proliferation and migration. These findings highlight the importance of the NPR3-raptor interaction in Musclin-mediated mTORC1 inactivation in VSMCs.

Several limitations also exist in this study. The mechanism by which the NPR3-raptor interaction affects mTORC1 activity remains unclear. We hypothesize that Musclin induces NPR3 to recruit and bind to raptor, causing a spatial conformational change in mTORC1. This isolates mTORC1 from the influence of upstream agonists. Alternatively, NPR3 may also competitively occupy the binding sites on mTORC1 for upstream agonists such as protein kinases, thereby inhibiting mTORC1 activity. These hypotheses require thorough experimental validation. Furthermore, vascular endothelial cells, fibroblasts, and inflammatory cells contribute to the development of IH. Whether Musclin affects other vascular cell types beyond VSMCs also needs further investigation.

In conclusion, this study demonstrated that skeletal muscle-derived Musclin alleviates vascular IH by inhibiting VSMC phenotypic switching. Additionally, AAV6-mediated Musclin overexpression in skeletal muscle suppresses mTORC1 activity through its specific receptor NPR3. Critically, Musclin induces direct binding between raptor, a core component of mTORC1, and NPR3, leading to mTORC1 inactivation and consequent inhibition of VSMC glycolysis and phenotypic switching.
